# PALLIATIVE AND END-OF-LIFE CARE FOR OLDER ADULTS WITH EXPERIENCES OF HOMELESSNESS

**DOI:** 10.1093/geroni/igad104.1190

**Published:** 2023-12-21

**Authors:** Kelly Melekis, Kelly Melekis

## Abstract

Although palliative and end-of-life (PEOL) care offers many benefits to those with life-limiting illness, unhoused older adults face significant barriers in accessing these types of care. This symposium examines disparities in PEOL care, emerging services for disadvantaged older adults, and recommendations for capacity-building within healthcare, housing, and aging services. The first paper is an exploratory study of programs serving the PEOL care needs of unhoused adults with serious illnesses. The second paper is a mixed methods study that explores provider perspectives on approaches and challenges to addressing the PEOL care needs among unhoused individuals. The third paper uses the CBPR method photovoice to highlight the experiences of terminally ill patients accessing social model hospice care, a free option to facilitate access to hospice for unhoused older adults. In addition to well-established disparities in the prevalence of serious illness and access to palliative care among people with experiences of homelessness, limits and barriers to holistic support remain once housed. The fourth paper uses secondary data analysis of the Research and Supportive Care at Later-life for Unhoused People (RASCAL-UP) study to describe current practices, perceived barriers, and recommendations in permanent supportive housing for older residents with complex comorbidities. The final paper, also using secondary data analysis of the RASCAL-UP study, addresses the role of skilled nursing for palliative care patients with histories of homelessness. Together, findings illuminate challenges to providing PEOL care for unhoused older adults and those with prior experiences of homelessness, as well as opportunities to improve access to care. This is a Hospice, Palliative and End-of-Life Care Interest Group Sponsored Symposium.

